# Domestic Violence as a Risk Factor for Postpartum Depression Among Ethiopian Women: Facility Based Study

**DOI:** 10.2174/1745017901814010109

**Published:** 2018-05-23

**Authors:** Addishiwet Fantahun Adamu, Yohannes Mehretie Adinew

**Affiliations:** 1 College of Health sciences, Addis Ababa University, Addis Ababa, Ethiopia; 2 College of Health sciences and Medicine, Wolaita Sodo University, Sodo, Ethiopia

**Keywords:** Depression, Postpartum depression, Maternal mental health and Ethiopia, Domestic, Domestic violence, Victims

## Abstract

**Background:**

Mental illness in women leads to an increased maternal morbidity and mortality. Postpartum depression accommodates various groups of depressive disorders and syndromes that occur within the first immediate year after delivery. Thus, this study aimed to assess the prevalence of postpartum depression symptoms and correlates among mothers attending public health centers of Addis Ababa, Ethiopia.

**Methods:**

Facility-based cross-sectional study was conducted on 618 women in their postpartum period. Simple random sampling technique was used to select three out of ten sub cities in Addis Ababa. Then, nine health centers were selected by lottery method from the three sub-cities. The number of women included from each health center was determined by proportional allocation. Study participants were enrolled by systematic random sampling. The Edinburgh Postnatal Depression Scale was used at a cutoff point >13 to detect depression. Descriptive statistics were done. The bivariate and multivariate analysis was also carried out to identify predictors of postpartum depression.

**Results:**

Significant proportion 144 (23.3%) of the women had the symptom of postpartum depression. Respondents who were the victims of domestic violence [AOR 3.1; 95% CI: 1.6-5.9], reported to have diagnosed with postpartum depression [AOR 4.41; 95% CI: 2.4-8.3], and dissatisfied with their marriage [AOR 2.9; 95% CI: 1.5-5.6] had higher odds of reporting postpartum depression symptoms.

**Conclusion:**

Postpartum depression is a common mental health problem during the postnatal period. Domestic violence was positively and significantly associated with the symptom of postpartum depression. Maternity services shall consider a sector that provides health care for women who encounter violence and develop symptoms of postpartum depression.

## 1 BACKGROUND

Postpartum Depression (PPD) explains various groups of depressive symptoms and syndromes that occur during the first immediate year after delivery [1]. It can be described by symptoms like lack of interest, low self-esteem, easily fatigability, sadness, sleep disturbance, loss of appetite [2], low concentration and inability to make decision, meaningless of life, negative attitude toward the baby and feeling of guilt and shame [3].

Globally, 8-19% of women have frequent symptoms of PPD [1]; this figure is higher in developing countries- 19.8% [2]. PPD is a major worldwide maternal mental health problem which leads to an increased maternal mortality, through adversely affecting physical health needs and more directly through suicide [4]. As a result, children’s growth and future life are tremendously affected by lack of mother-child bonding, lack of breastfeeding, and poorer care [2].

Basically, almost all women are at risk of mental health problems during pregnancy and postnatal period in the immediate first year after delivery. Among the factors that lead women to develop postpartum depression, domestic violence is the major [5]. Violence has devastating consequences on women's physical, psychological, mental and reproductive health: disability (up to loss of life), unwanted pregnancy, depression, loss of confidence and sexually transmitted diseases to mention few [6, 7]. These consequences of violence further have an emotional impact and are also linked to negative health behaviors, such as substance abuse and mood disorders like mania and depression [8, 9].

Despite its disastrous effects especially in low income countries, PPD is not understood well [10]. Therefore, it is mandatory to have an insight into and a plan for the implementation of strategies on prevention and early identification of the problem. Thus, the study was aimed to assess the prevalence and factors associated with the symptom of postpartum depression among mothers attending public health centers in Addis Ababa, Ethiopia.

## 2 METHODS

### 2.1 Study Design and Area

Facility-based cross-sectional study was employed in selected health centers of Addis Ababa city, the capital of Ethiopia, from Jan to Feb 2017. Addis Ababa has a total population of 3,048,631; of which 52.3% are females [11]. The city has a total of 11 public hospitals, 90 health centers, 31 private hospitals and 700 different level private clinics [12]. The study populations were women who came to sampled health centers for postnatal care and vaccination services within the first six weeks after delivery.

### 2.2 Sample Size Determination and Sampling Procedure

The sample size was determined using single population proportion formula. Based on a significance level of 95%, 4% margin of error, 19.8% prevalence of PPD among study subjects [2], 10% for possible nonresponse and 1.5 design effect, making the total sample size 629.

Multistage sampling technique was employed to select the respondents. Three sub cities namely Bole, Kolfe keranio and Yeka were randomly selected by names from hat method out of the ten sub-cities of Addis Ababa city administration. Then, nine health centers, three from each of the sampled three sub-cities, were selected by lottery, names from hat method. The number of women included in the study from the selected health centers was determined using the proportion to size allocation technique on the basis of previous three-month data from the respective health centers. Participants were enrolled to the study by systematic random sampling technique. To identify the interval, the average number of women expected per day in each health center was divided by the number of women to be interviewed per day from respective health centers. The first woman was selected by lottery method and then every other (second) woman (calculated for each health center) visiting the health centers was enrolled to the study. In case a woman we approached refused, we enrolled the next immediate woman.

### 2.3 Variables of the Study

#### 2.3.1 Dependent Variable

Postpartum depression

#### 2.3.2 Independent Variable

Socio-demographic characteristics, social support, obstetric factors and psychiatric history

### 2.4 Operational Definitions

#### 2.4.1 Postpartum Depression Symptoms

According to Edinburgh Postnatal Depression Scale (EPDS) questions 1, 2, & 4 were scored 0, 1, 2 and 3 with the first choice scored as 0 and the last choice scored as 3. Questions 3, 5-10 are reversely scored, with the first choice scored as 3 and the last choice scored as 0. After adding up all the scores, those women who scored >13 were defined to have symptoms of postpartum depression [13, 14].

#### 2.4.2 Postpartum Period

A period beginning immediately after the birth of a child and extending to the sixth week.

#### 2.4.3 Social Support

Social support was measured using the Maternity Social Support Scale (MSSS) developed by Webster and colleagues [15]. The scale contains six items and includes questions on family support, friendship network, help from spouse, conflict with spouse, feeling controlled by spouse, and feeling unloved by spouse. Each item was measured on a five-point Likert scale and a total score of 30 was possible. We classified social support into two categories; high social support (for scores 18–30) and low social support (below 18) categories. The internal consistency of the scale was tested using Cronbach’s alpha and was found to be 0.74.

#### 2.4.4 Domestic Violence

It is defined as being the victim of domestic violence during perinatal period if she reported any of the following experiences with a husband or a partner: having something thrown at her; being pushed, or slapped, or kicked, or beaten up; being threatened or attacked with any weapon; being physically forced to have sex and/or perform any sexual act when not have the desire.

#### 2.4.5 Data Collection Tool and Procedures

A structured interviewer-administered questioner was used to collect data. The instrument was adopted from previous researches [10, 16-22]. The questioner was designed in English and translated to local language, Amharic and then back to English by a third person to cheek for internal consistency. The tool composed of two sections; the first section involved four parts. Part one had 15 questions about socio-demographic characteristics, part two had 11 questions concerning obstetric factors, part three had 2 questions related to past psychiatric history and part four involved 6 items on social support. Section two had 10 questions on EPDS that indicates how the mother has felt during the previous 7 days. The EPDS is widely used and has been validated for use in different countries and settings [23-27], including urban and rural part of Ethiopia [25, 28] to identify postnatal depression symptoms and has generated sensitivity and specificity of 78.9% and 75.3% respectively [28]. Thus, it is recommended that the EPDS be used in routine postnatal screening [29]. Five diploma nurses, who were not the employees of the selected health centers, collected the data.

#### 2.4.6 Data Quality Control Management

Appropriate training and supervision was given to data collectors. The instrument was pretested in Arada health center on 32 postnatal women and necessary revisions, and sequence rearrangement of questions to remove the redundant ones, were made prior to the actual data collection.

#### 2.4.7 Data Analysis Procedure

Filled data were checked for completeness and entered to Epi data and then exported to SPSS version 21 for further analysis. Descriptive statistical analysis was carried out. Both bivariate and multivariate logistic regression models were also carried out to identify associated factors. Odds ratios and their 95% confidence intervals were computed and variables with p value < 0.05 were considered significant.

## 3 RESULTS

### 3.1 Socio-Demographic Characteristics

A total of 618 post-partum women responded fully to all the questions out of 629 requested to participate, yielding a response rate of 98.2%. The number of participants included in the study was 197(32%) form Bole, 229(37%) from Kolfe keranio and 192(31%) from Yeka sub-cities**.** The median age of the respondents was 28 years (range; 16-46 years). Majority (526, 85.1%) were married, while 502(81.2%) of the participants had attended formal education. Two hundred eighty-one (45.5%) of the respondents were housewives (Table **1**).

### 3.2 Obstetric and Clinical Characteristics

More than a third (228, 36.9%) of the participants reported that it was their first pregnancy; and for 179 (29%) women, the pregnancy was unplanned. About one-fifth (136, 22.0%) of the respondents were not happy with the sex of their last baby. The prevalence of caesarian delivery was 131 (21.2%). Furthermore, one-fourth of the participants 164(26.5%) had suffered from illnesses (obstetric and non-obstetric) for which they had sought treatment, during their pregnancy. It was also reported that 21(3.4%) of the respondents’ recent pregnancy was eventfull (Table **2**).

### 3.3 Personal and Family history of Depression and Social Support Among the Women

Among women who had previously given birth, 93 (23.8%) of them reported that they had been diagnosed with PPD. In addition, 90 (14.6%) of the respondents had a family history of depression. Eighty-seven (14.1%) of the study participants reported they had experienced domestic violence. Nearly one-fifth (113,18.8%) of the women described their relationship with their husband as unsatisfactory and 104 (16.8%) of the respondents reported not being enough social. Regarding the relationship they had with their mother in law, 178 (28.8%) stated that they were unhappy (Table **3**).

### 3.4 Prevalence of Postpartum Depression Symptoms

A significant proportion of 144(23.3%) women had symptoms of postnatal depression; the score ranged from 1 to 28 in the overall sample. Fifty-one (8.3%) of the respondents scored 1 while only two (0.3%) scored 28.

### 3.5 Edinburgh Postnatal Depression Scale (EPDS) Responses Among Participants

About one-tenth (69,11.2%) reported that they were not able to laugh and see the funny side of things. For forty-eight (7.8%) of the participants, it was so difficult to look forward with enjoyment to things. Nearly one-tenth (58, 9.4%) of the women were blaming themselves unnecessarily. Nearly one-sixth of the study participants were anxious or worried for no good reason. In addition, 25(4.0%) stated that they couldn’t be able to cope up with things at all. For twenty-six (4.2%) of the study participants, it was difficult to sleep most of the time. In addition, 18(2.9%) were unhappy and have been crying most of the time and only 2 (0.35%) had a thought of harming themselves (Table **4**).

### 3.6 Factors Associated with Symptoms of Postpartum Depression

The result of the multivariate analysis showed that domestic violence, previous history of mental health problem and dissatisfaction with the relationship were positively associated with symptoms of postnatal depression.

Domestic violence was found to affect postnatal depression. Respondents who experienced domestic violence had three [AOR: 3.1, 95% CI: 1.6, 5.9] times the odds of reporting symptoms of postpartum depression in contrast to those who had no history of domestic violence. Similarly, participants who were unhappy about their relationship had about three [AOR: 2.7, 95% CI: 1.4, 5.2] times the odds of developing symptoms of postpartum depression than their counterparts. Furthermore, the previous history of postpartum depression was also found to have an association with the re-attack of postpartum depression. Respondents who had been previously diagnosed for depression had four [AOR: 4.2, 95% CI: 2.3, 7.8] times higher odds of reporting symptoms of depression than those who had no previous history (Table **5**). Variables such as educational status, occupational status, mode of delivery, relationship with mother-in-law and sex of the newborn didn’t show any association with symptoms of postpartum depression.

## 4 DISCUSSION

Reproductive age women are vulnerable to mental health problems especially during pregnancy and postpartum period as a result of domestic violence [30, 31]. This study also certifies the role of intimate partner violence on postpartum depression among postnatal women to be significant along with the previous history of mental health problem and dissatisfaction in marriage.

Significant proportion of women i.e 144 (23.3%) women experienced symptoms of postpartum depression implying that the problem is now becoming a substantial concern for which services are urgently needed. This was somewhat comparable with studies from China 27.37% [16], City of Poland 23.2% [21] and Lahore 25% [32]. However this figure was higher compared to findings from Japan 7.7% [33], Canada 8.69% [34], Qatar 18.6% [35] Turkey 15.4% [36] and Sudan 9.2% [37]. This discrepancy might be explained by different tools, assessment period and methods used. For instance, the study in Japan was conducted by using Japanese version of 12 questions which assessed the general health status of participants at fourth month after delivery by taking ≥4 as cut-off score. Whereas in a study from Qatar, depression anxiety stress scale was used for face to face interview. On the other hand, similar studies from Ethiopia found a higher prevalence report than the present study 32.8% [38] in Amhara region and 31.5% [39] in Oromia region. Likewise, studies from Iran [40], South Africa [19] and Uganda [41] also reported an elevated prevalence of symptoms of postpartum depression.

As observed from this and other studies, it was evident that domestic violence was a major determinant factor of postpartum depression [AOR: 3.1, 95% CI: 1.6, 5.9]. Despite variability in methodology and definitions of abuse, findings from different parts of the world showed a strong relationship between abuse and risk of postnatal depression. In this study violences were typically physical and verbal (insulting) abuses. A systematic review of literature also revealed a significant association between abuse and PPD [42]. In addition, other studies from Canada [43], China [44], Chili [45] and Pakistan [46] stated that participants with some sort of intimate partner abuse before or during pregnancy presented with the symptom of postpartum mental health problems. In a cohort study from Iran [47], women who were screened positive for postnatal depression had been abused.

Moreover, participants who had previously diagnosed with PPD were found to have higher odds [AOR: 4.2, 95% CI: 2.3, 7.8] of reporting the symptom of postnatal depression. This substantial higher risk of postpartum depression was concordance with many other findings which reported a magnified likelihood of depression among individuals who had a previous history of mental health problem [33, 34, 48, 49]. Furthermore, a study from Brazil [22] revealed that postpartum depression was not only related with the personal history of mental health problem but also a family history of depression. This finding was again supported by another study which was conducted on pregnant women in Addis Ababa, Ethiopia [50]. Stressful moment of pregnancy and delivery could be the reason for relapse of depression among women who had previously diagnosed with PPD. Hormonal imbalance during pregnancy could also be another reason.

In the present study, 113(18.8%) of participants were unhappy with their marriage and discovered that women who had a deprived relationship with their partners had higher scores of postpartum depression symptoms [AOR: 2.7, (95% CI: 1.4, 5.2]. Likewise, studies carried out on Ugandan [41] and Iranian [51] women were also in agreement with this finding; loss of satisfaction in marriage would be a contributing factor to the symptom of postpartum depression. Besides, a literature reviewed from seventeen studies with a total of 19,132 Arab women found unsatisfactory relationship to be a significant risk factor for mental health problem in postpartum period [52]. Constantly this problem was also highlighted by another literature review conducted on similar study subjects [53].

## 5 LIMITATIONS OF THE STUDY

The study was limited to six weeks postpartum; the persistence of depression symptoms beyond those weeks of postnatal period was not considered, plus since sexual violence and mental illness are sensitive topics, it is very prone to reporting bias, in most cases, women tend to under-report; thus the prevalence might be somehow underestimated. This study has shared the limitations of cross-sectional studies, the difficulty in determining causal relationships between variables.

## AUTHORS CONTRIBUTIONS

AF: Initiation of the study, Design, Implementation, analysis and Write-up; YMA: Design, Analysis and Write-up as well as prepared the manuscript for publication. All authors read and approved the final manuscript.

## CONCLUSION

Domestic violence is positively associated with the symptom of postpartum depression. Thus, counseling in maternity services should consider the mental disorder-related healthcare needs of those who experienced violence. Further large prospective cohort studies are warranted to identify the relationship between violence and depression.

## Figures and Tables

**Table 1:** Socio-Demographic characteristics among women in postpartum period, in health centers of three sub-cities of Addis Ababa, Ethiopia (N=618).

**Characteristics**	-	**Depression**	
-	-	**Yes**	**No**
**Age in years**	15-24	32 (23.1)	106 (76.9)
-	25-34	95 (15.2)	315 (32.1)
-	>35	17 (2.9)	53 (5.9)
**Marital status**	Married	122 (19.8)	404 (65.3)
-	Unmarried	22 (3.5)	70 (11.4)
**Attended school**	Yes	100(19.9)	402(80.1)
-	No	44(37.9)	72(62.1)
**Level of education (502)**	Primary school	39 (23.9)	82 (76.1)
-	Secondary school	23 (21.4)	84(78.6)
-	Technical or vocational	6 (10.5)	51(89.5)
-	Diploma	25 (22.4)	87(77.6)
-	Degree and above	22 (20.9)	83(79.1)
**Occupational status**	Employed	67(19.9)	270(80.1)
-	Housewife	77(27.4)	204(72.6)
**Difficult with income**	Yes	71 (33.8)	139(66.2)
-	No	73 (17.8)	335(82.1)
**Monthly average income**	<445	47 (27.6)	123(72.6)
-	446-1200	8 (12.5)	56(87.5)
-	1201-2500	15 (15.4)	82(84.6)
-	2501-3500	15 (15.6)	81(84.4)
-	>3501	59 (30.8)	132(69.1)

**Table 2:** Participants Obstetrics and clinical characteristics in postpartum period, from health centers of three sub-cities of Addis Ababa, Ethiopia (N= 618).

**Characteristics**		**Depression**	
		Yes	No
**Number of pregnancy**	1	33(14.4)	195(85.6)
	2-3	82(26.3)	229(73.7)
	≥4	29(39)	50(63.2)
**Planed pregnancy**	Yes	87(19.8)	352(80.2)
	No	57(31.8)	122(68.2)
**Sex of last baby**	Male	80(26)	227(74)
	Female	64(20.5)	247(79.5)
**Desired sex of the baby**	Undesired	30(22.1)	106(77.9)
	Desired	36(17.4)	171(82.6)
	I don’t mind	78(28.4)	197(71.6)
**Mode of delivery**	Vaginal	99(23.8)	317(76.2)
	Instrumental delivery	25(35.2)	46(64.8)
	Cesarean section	20(15.3)	111(84.7)
**Illness during pregnancy**	Yes	43(26.2)	121(73.8)
	No	101(22.2)	353(77.8)
**Experience death of a baby**	Yes	19(47.5)	21(52.5)
	No	125(21.5)	453(78.5)
**Hospitalized child**	Yes	32(30.7)	72(69.3)
	No	112(21.7)	402(78.3)
**Stressful life event during pregnancy**	Yes	6(28.5)	15(71.5)
	No	138(23.1)	459(76.9)

**Table 3:** Personal and family history of depression and social support among postpartum women’s, from health centers of three sub-cities of Addis Ababa, Ethiopia (N= 618).

**Characteristics**	-	**Depression**	
		**Yes**	**No**
**Previous history of depression**	**Yes**	53(57.0)	40(43.0)
	**No**	91(17.3)	434(82.7)
**Relatives suffered from mental illness**	**Yes**	33(36.6)	57(63.4)
	**No**	111(21.0)	417(79.0)
**Abuse/domestic violence**	**Yes**	52(59.8)	35(40.2)
	**No**	92(17.3)	439(82.7)
**Satisfied with marriage**	**Yes**	77(15.2)	428(84.8)
	**No**	67(59.3)	46(40.7)
**Social support**	**Yes**	64(12.4)	450 (87.6)
	**No**	80(76.9)	24 (23.1)
**Relatives present during labor**	**Yes**	123(21.9)	437 (78.1)
	**No**	21(36.2)	37 (63.8)
**Satisfied in relation with mother-in-law**	**Yes**	88(20.0)	352(80.0)
	**No**	56(31.5)	122(68.5)

**Table 4:** Edinburgh postnatal depression scale responses among postpartum women’s, from health centers of three sub-cities of Addis Ababa, Ethiopia (N= 618).

**Characteristics**	**Frequency**	**Percentage**
**Experienced laugh and see funny side of things**		
**As much as always I could**	394	63.8
**Not quite so much now**	102	16.5
**Definitely not so much now**	53	8.6
**Not at all**	69	11.2
**Look forward with enjoyment to things**		
**As much as I ever did**	385	62.3
**Rather less than I used to**	125	20.2
**Definitely less than I used to**	60	9.7
**Hardly at all**	48	7.8
**Blamed yourself unnecessarily**		
**No never**	309	50.0
**Not very often**	119	19.3
**Yes some of the time**	132	21.4
**Yes most of the time**	58	9.4
**Been anxious or worried for no good reason**		
**No not at all**	319	51.6
**Hardly ever**	70	11.2
**Yes sometimes**	175	28.3
**Yes very often**	54	8.7
**Felt scared or panic for no good reason**		
**No not at all**	375	60.7
**No, not much**	102	16.5
**Yes, sometimes**	111	18.0
**Yes, quite a lot**	30	4.9
**Things have been on top of you**		
**No I have been coping**	388	62.8
**No most of the time**	166	18.8
**Yes sometimes I haven’t been coping as well as usual**	89	14.4
**Yes most of the time I haven’t been able to**	25	4.0
**Difficult to sleep**		
**No, not at all**	375	60.7
**Not, very often**	144	23.3
**Yes sometimes**	73	11.8
**Yes most of the time**	26	4.2
**Felt sad or miserable**		
**No, not at all**	386	62.5
**Not, very often**	149	24.1
**Yes sometimes**	62	10.0
**Yes most of the time**	21	3.4
**So unhappy you have been crying**		
**No, never**	410	63.3
**Only occasionally**	149	24.1
**Yes quite often**	41	6.6
**Yes, most of the time**	18	2.9
**Thought of harming your self**		
**Never**	544	88.0
**Hardly ever**	41	6.6
**Sometimes**	31	5.0
**Yes, quite often**	2	0.3

**Table 5:** Bivariate and multivariate logistic regression analysis output of factors associated with postpartum depression among postpartum women from health centers of three sub-cities of Addis Ababa, Ethiopia (N=618).

**Variables**		**Depression**		**Crude OR** **[95% CI]**	**Adjusted OR** **[95% CI]**
		**Yes**	**No**		
**Attended School**	Yes	100(19.9)	402(80.1)	1	1
	No	44(37.9)	72(62.1)	2.5(1.6,3.8)	0.8(0.4,1.5)
**Occupation**	Employed	67(19.9)	270(80.1)	1	1
	Unemployed	77(27.4)	204(72.6)	1.5(1.0,2.2)	1.0(0.6,1.8)
**Mood of Delivery**	CS	20(15.3)	111(84.7)	0.6(0.3,1.0)	0.5(0.2,1.0)
	Instrumental	25(35.2)	46(64.8)	1.7(1.0,3.0)	0.8(0.4,1.8)
	Vaginal	99(23.8)	317(76.2)	1	1
**History of Depression**	Yes	53(57.0)	40(43.0)	**6.3(4.0,10.1)**	4.2(2.3,7.8)**
	No	91(17.3)	434(82.7)	**1**	**1**
**Domestic Violence**	Yes	52(59.8)	35(40.2)	**7.1(4.4,11.5)**	3.1(1.6,5.9)**
	No	92(17.3)	439(82.7)	**1**	**1**
**Happy in Marriage**	Yes	77(15.2)	428(84.8)	**1**	1
	No	67(59.3)	46(40.7)	**8.1(5.2,12.7)**	2.7(1.4,5.2)**
**Relation With Mother-in-law**	Happy	88(20.0)	352(80.0)	1	1
	Not happy	56(31.5)	122(68.5)	1.8(1.2,2.7)	0.8(0.5,1.5)
**Desired Sex of Newborn**	Undesired	30(22.1)	106(77.9)	0.7(0.4,1.2)	1.0(0.5,2.0)
	Desired	36(17.4)	171(82.6)	0.5(0.3-0.8)	1.0(0.6,1.8)
	I don’t mind	78(28.4)	197(71.6)	1	1

CS: Cesarean section; Instrumental: Instrumental delivery

## LIST OF ABBREVIATIONS

EPDS: = Edinburgh Postnatal Depression Scale
PPD: = Postpartum Depression

## AVAILABILITY OF DATA AND MATERIALS

Data supporting this finding are available and can be accessed with reasonable inquiry.
